# Pandemic-driven shift: increase in silver diamine fluoride utilization among Medicaid-enrolled children during the COVID-19 public health emergency

**DOI:** 10.3389/fpubh.2025.1546365

**Published:** 2025-03-25

**Authors:** Beau D. Meyer, Carla Shoff, Natalia I. Chalmers

**Affiliations:** ^1^Division of Pediatric Dentistry, The Ohio State University College of Dentistry, Columbus, OH, United States; ^2^Office of the Administrator, Centers for Medicare & Medicaid Services (CMS), Baltimore, MD, United States

**Keywords:** health services accessibility, pediatric dentistry, healthcare disparities, silver diamine fluoride, reimbursement policy in health care

## Abstract

**Introduction:**

Untreated dental caries remains a significant public health issue, particularly among children and adolescents from low-income families, where disparities persist. The COVID-19 public health emergency (PHE) changed dental care practices, leading to an increased focus on minimally aerosolizing treatments such as silver diamine fluoride (SDF). This study aimed to describe the temporal changes in SDF utilization among Medicaid-enrolled children across the United States before and during the first half of the COVID-19 PHE. Additionally, the study examined the impact of demographics and state-level policies on SDF utilization.

**Methods:**

We conducted a multiyear cross-sectional study using enrollment and claims data from the Transformed Medicaid Statistical Information System (T-MSIS) for 2019, 2020, and 2021. The study population included Medicaid and Children’s Health Insurance Program (CHIP) beneficiaries aged <21 years. We analyzed SDF utilization rates and compared them with other dental services, stratifying the data by age, sex, race/ethnicity, and rurality. Multilevel logistic regression models were used to identify significant predictors of SDF utilization.

**Results:**

The study included approximately 39 million children each year. SDF utilization per 1,000 enrollees increased from 9.10 in 2019 to 16.81 in 2021, with the most significant increases observed in children aged 0–6 years, those living in rural areas, and American Indian/Alaskan Native children. The state-level reimbursement policy for SDF was the most significant predictor, with children in states with such policies being 10.5 times more likely to receive SDF treatment.

**Conclusion:**

The COVID-19 PHE significantly impacted SDF utilization among Medicaid-enrolled children, highlighting the importance of state-level policies. The findings can be used to develop targeted approaches for clinicians to improve access to SDF treatment to address oral health disparities.

## Introduction

Untreated dental caries is a significant public health problem. Despite reductions in untreated caries in children aged 2–5 years, minimal change has been observed for older children and adolescents (1). Significant oral health disparities persist for children aged 2–5 years. One-third of Mexican American and 28% of non-Hispanic Black children have had cavities in their primary teeth, compared with 18% of non-Hispanic White children (2). Children and adolescents from families with incomes <100% of the federal poverty guidelines are about twice as likely to have untreated tooth decay as their peers from families with incomes >200% of the federal poverty guidelines (1, 3).

Traditional dental treatment includes surgical high-speed handpieces, which are aerosol-generating. The dental care system’s operations were significantly impacted by the COVID-19 public health emergency (COVID-19 PHE) driven by a virus transmitted through air as an aerosol. During the COVID-19 PHE, many state dental boards implemented policies that temporarily closed dental practices and/or restricted dental care to treatments that did not generate aerosols (4). Silver diamine fluoride (SDF) emerged as a minimally aerosolizing alternative to traditional treatment. SDF was first introduced in the United States in 2014. The U.S. Food and Drug Administration (FDA) has classified SDF as a Class II medical device, meaning there is some intermediate-level of risk to the patient. SDF is cleared for use in the treatment of tooth sensitivity, which is the same type of clearance as fluoride varnish and must be professionally applied (5). SDF is recommended as part of an ongoing caries management plan to optimize individualized patient care consistent with the goals of a dental home, and its use may prevent or delay the need for more extensive and expensive procedures (6). Teeth treated with SDF have a notable black tooth discoloration following the treatment (5, 6). Application protocols recommend careful follow-up and re-application at least every 6 months unless disease progresses or parental preferences change (5, 6).

Other studies have examined how the pandemic altered dental utilization patterns (7–11). Specifically, dental treatment and claims volumes for publicly and privately insured children were significantly lower in 2020 than in 2019, but by 2021, volumes had returned to or approached previous volumes (7). One study of commercial claims found higher dental expenditures per child during the initial wave of the pandemic for children ages 0–5 (8). Publicly available annual dental visit data obtained from Centers for Medicare & Medicaid Services (CMS) Early and Periodic Screening, Diagnostic, and Treatment (EPSDT) reports (Form CMS-416) also show precipitous drops in use in 2020 compared to 2018 and 2019 (12). Still, a rebound in 2021 is consistent with other studies.

The primary objective of this study was to describe temporal changes in SDF utilization before and during the first half of the COVID-19 PHE. Specifically, we examined and described variations in SDF utilization among Medicaid-enrolled children at the state level with overall dental utilization. In a secondary analysis, we determined the association between state policy for SDF reimbursement and SDF utilization.

## Materials and methods

### Data sources

This multiyear cross-sectional study used enrollment and claims files from the 2019–2021 Transformed Medicaid Statistical Information System (T-MSIS) Analytic Files (TAF) maintained by CMS. Specifically, the Demographic and Eligibility and Other Services files were utilized and were accessed through the CMS Chronic Conditions Data Warehouse (CCW) (13). The Economic Research Service Rural–Urban Commuting Area Codes were used to determine the rural/urban status of the beneficiary’s residence (14). This data was linked to the T-MSIS data by the beneficiary’s ZIP code. The study was covered by the Common Rule exemption, 45 CFR 46.104(d) (4)(iv), and did not require institutional review board review because it was not considered human participant research.

### Study population

This study included Medicaid and Children’s Health Insurance Program (CHIP) beneficiaries under 21 years old who are non-dually eligible for Medicare each calendar year. The years included for analysis were 2019, 2020, and 2021. To be included in the study, beneficiaries were required to live in a state not identified as having data quality concerns, according to the CMS DQ Atlas (15). States assigned as high concern or unusable data on the following topics were excluded from all analyses: claims volume and professional services procedure codes. Beneficiaries from 5 states were excluded from all analyses (MA, MN, NJ, RI, UT). Beneficiaries from 22 states with high concern or unusable race and ethnicity data were excluded from all analyses stratified by race and ethnicity, 3 of which were already excluded for claims data quality concerns (AL, AR, AZ, CO, CT, DC, HI, IA, KS, LA, MA, MD, MO, MT, NY, OR, RI, SC, TN, UT, WV, WY).

### Variables and outcomes

Demographic data such as age, sex, and race/ethnicity (in select states) were collected from the Demographic and Eligibility files. Children were categorized into one of three age groups to approximate the stages of developing dentition: 0–6 years (e.g., primary dentition), 7–11 years (e.g., mixed dentition), and 12–20 years (e.g., permanent dentition). Race and ethnicity was not available in all states, but when available, it was collected as the following categories: American Indian/Alaskan Native, Asian/Pacific Islander, Hispanic, Non-Hispanic Black, Non-Hispanic White, Multiracial/Other Race/Unknown. Rurality was assigned by the beneficiary’s ZIP Code contained in the eligibility/enrollment files and dichotomized into rural versus urban according to the Economic Research Service Rural–Urban Commuting Area Codes (14).

**Figure 1:**
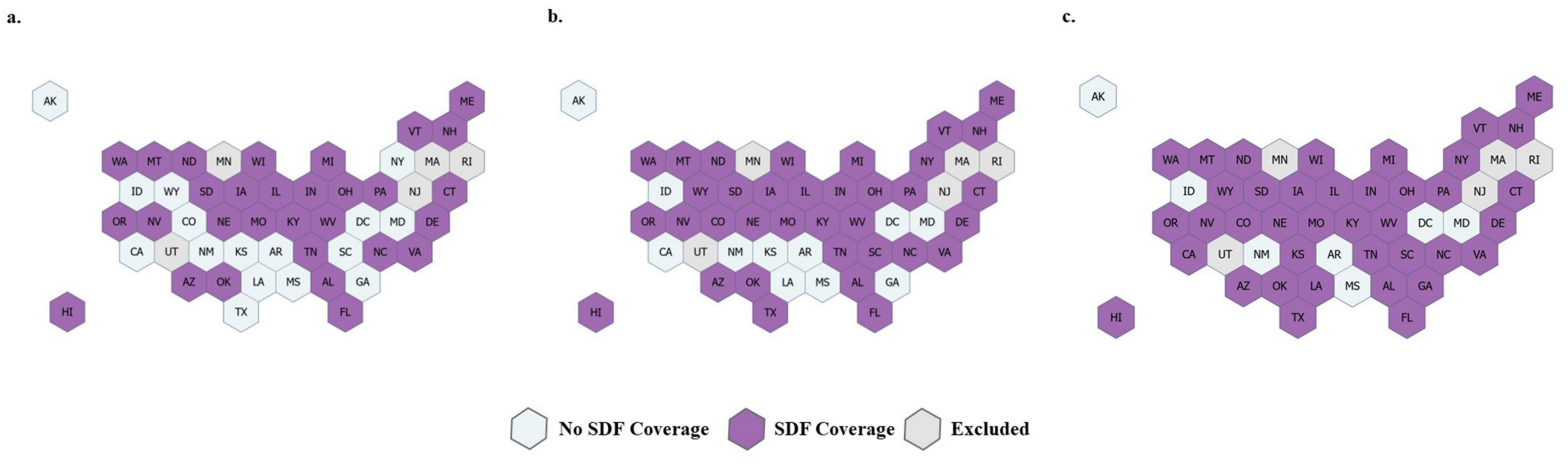
State Medicaid coverage of D1354 or D1355 (SDF coverage) based on publicly available fee-for-service fee schedules, provider manuals, and provider bulletins in **(a)** 2019, **(b)** 2020, and **(c)** 2021. Five states were excluded due to data quality issues.

For the SDF coverage policy, there are no single resource documents when state Medicaid agencies approve a reimbursement policy for SDF (CDT code D1354 or D1355). To determine the year each state implemented reimbursement for D1354 or D1355, the research team reviewed publicly available provider manuals, fee-for-service fee schedules, and provider bulletins for each state Medicaid program. We did not examine state-specific managed care plans for D1354 coverage, so our coverage designations are limited only to the fee-for-service elements of state programs (Figure 1). The two primary dental utilization outcomes were as follows: (1) at the state level, we calculated the rate of children who had any dental visit. The numerator was set to the unduplicated number of children with CDT codes D0000-D9999, and the denominator was all enrolled non-dual eligible children. The outcome was reported as the number of children per 1,000 Medicaid and CHIP enrolled children per year who had any dental visit. (2) Second, among those children who had a dental visit, we calculated the rate for those who had an SDF application according to CDT codes D1354 or D1355. The outcome was reported as the number of children per 1,000 Medicaid and CHIP enrolled children with a dental visit who received an SDF application.

### Statistical analysis

Descriptive statistics were calculated at the state level and overall for each outcome and year, stratified by age group, sex, rurality, and coverage status. Chi-square tests were used to test for significant differences in the rates across categories within each group and whether the category-specific rates significantly differ across years. A multilevel logistic regression model was used to predict the odds of SDF for 1 year of data (2021). The state FIPS code is included in the model as a level-two random intercepts parameter to adjust for the similarity of beneficiaries residing within the same state. Statistical significance was set at *p* ≤ 0.05; all *p*-values were 2-tailed. Analyses were conducted with SAS Enterprise Guide 7.1 (SAS Institute, Inc.; Cary, NC, United States) and Stata version 18.0 (StataCorp LLC.; College Station, TX, United States).

## Results

Approximately 39 million children were included in each analysis year. A full demographic description is in Table 1. The majority were males and lived in urban counties. The number of states with an SDF reimbursement policy increased from 2019 to 2021, and subsequently, the number of children living in states with these policies nearly doubled (20.4 million in 2019 to 36.8 million in 2021). The rate of children with any dental visit significantly decreased from 2019 (443.32 per 1,000 beneficiaries) to 2020 (401.74 per 1,000 beneficiaries) but rebounded in 2021 (444.26 per 1,000 beneficiaries) (Table 2). The same utilization patterns were noted when stratified by age, sex, race when available, and rurality. However, SDF utilization was different. In each year across the study period, SDF utilization significantly increased from 2019 (9.10 per 1,000 beneficiaries who had a dental visit) to 2020 (12.02 per 1,000 beneficiaries who had a dental visit) to 2021 (16.81 per 1,000 beneficiaries who had a dental visit) (Table 3).

**Table 1:** Beneficiary characteristics of Medicaid/CHIP beneficiaries ages 0 to 20^a^.

	Beneficiaries		
	2019	2020	2021
	No. (rate)	No. (rate)	No. (rate)
Overall	39,436,293 (1000.00)	38,107,587 (1000.00)	39,255,737 (1000.00)
Age groups			
Age 0 to 6	14,081,971 (357.08)	13,203,418 (346.48)	12,987,546 (330.84)
Age 7 to 11	9,887,532 (250.72)	9,524,565 (249.94)	9,764,051 (248.73)
Age 12 to 20	15,466,790 (392.20)	15,379,604 (403.58)	16,504,140 (420.43)
Sex			
Female	19,409,676 (492.18)	18,744,801 (491.89)	19,264,942 (490.75)
Male	20,026,617 (507.82)	19,362,786 (508.11)	19,990,795 (509.25)
Race and ethnicity^b^			
American Indian/Alaskan Native	387,979 (9.84)	350,561 (9.20)	364,032 (9.27)
Asian/Pacific Islander	912,205 (23.13)	870,086 (22.83)	895,956 (22.82)
Non-Hispanic Black	5,473,485 (138.79)	5,343,444 (140.22)	5,443,749 (138.67)
Hispanic	8,468,496 (214.74)	8,130,713 (213.36)	8,332,722 (212.27)
Non-Hispanic White	9,075,230 (230.12)	8,916,802 (233.99)	9,102,981 (231.89)
Multiracial/Other Race/Unknown	2,897,690 (73.48)	3,054,949 (80.17)	3,344,343 (85.19)
Residence designation			
Rural	7,141,818 (181.10)	6,934,993 (181.98)	7,156,063 (182.29)
Urban	32,294,475 (818.90)	31,172,594 (818.02)	32,099,674 (817.71)
State dental coverage status (D1354 or D1355)			
No coverage	19,043,690 (482.90)	10,071,587 (264.29)	2,411,334 (61.43)
Coverage	20,392,603 (517.10)	28,036,000 (735.71)	36,844,403 (938.57)

^aBeneficiaries from 5 states were excluded due to data quality concerns.^

^bAnalyses stratified based on race and ethnicity excluded 19 additional states due to data quality concerns.^

**Table 2:** Dental visits counts and rates per 1,000 Medicaid/CHIP beneficiaries ages 0 to 20^a^ stratified by baseline characteristics.

	Beneficiaries with a dental visit			*p*-value^c^
	2019	2020	2021	
	No. (rate)	No. (rate)	No. (rate)	
Overall	17,482,904 (443.32)	15,309,231 (401.74)	17,439,678 (444.26)	<0.001
Age groups				
Age 0 to 6	5,400,391 (383.50)	4,515,952 (342.03)	5,059,668 (389.58)	<0.001
Age 7 to 11	5,456,070 (551.81)	4,735,182 (497.15)	5,334,691 (546.36)	<0.001
Age 12 to 20	6,626,443 (428.43)	6,058,097 (393.90)	7,045,319 (426.88)	<0.001
Sex				
Female	8,768,442 (451.76)	7,725,525 (412.14)	8,776,050 (455.55)	<0.001
Male	8,714,462 (435.14)	7,583,706 (391.66)	8,663,628 (433.38)	<0.001
Race and ethnicity^b^				
American Indian/Alaskan Native	167,091 (430.67)	126,647 (361.27)	145,712 (400.27)	<0.001
Asian/Pacific Islander	419,802 (460.21)	349,223 (401.37)	409,222 (456.74)	<0.001
Non-Hispanic Black	2,174,532 (397.28)	1,917,127 (358.78)	2,181,414 (400.72)	<0.001
Hispanic	4,279,376 (505.33)	3,703,152 (455.45)	4,098,815 (491.89)	<0.001
Non-Hispanic White	3,766,586 (415.04)	3,379,648 (379.02)	3,827,466 (420.46)	<0.001
Multiracial/Other Race/Unknown	1,066,155 (367.93)	1,012,743 (331.51)	1,220,712 (365.01)	<0.001
Residence designation				
Rural	3,216,721 (450.41)	2,793,998 (402.88)	3,200,023 (447.18)	<0.001
Urban	14,266,183 (441.75)	12,515,233 (401.48)	14,239,655 (443.61)	<0.001
State dental coverage status (D1354 or D1355)				
No coverage	9,411,259 (494.19)	4,028,403 (399.98)	1,290,018 (534.98)	<0.001
Coverage	8,071,645 (395.81)	11,280,828 (402.37)	16,149,660 (438.32)	<0.001

^aBeneficiaries from 5 states were excluded due to data quality concerns.^

^bAnalyses stratified based on race and ethnicity excluded 19 additional states due to data quality concerns.^

^cχ2 tests were used to test statistical significance of utilization rates among categorical variables in 2019, 2020, and 2021.^

**Table 3:** Silver diamine fluoride counts and rates per 1,000 Medicaid/CHIP beneficiaries ages 0 to 20^a^ *with a dental visit* stratified by baseline characteristics.

	Beneficiaries with SDF treatment			*p*-value^c^
	2019	2020	2021	
	No. (rate)	No. (rate)	No. (rate)	
Overall	159,146 (9.10)	184,089 (12.02)	293,243 (16.81)	<0.001
Age groups				
Age 0 to 6	87,773 (16.25)	100,115 (22.17)	152,160 (30.07)	<0.001
Age 7 to 11	50,359 (9.23)	55,180 (11.65)	93,274 (17.48)	<0.001
Age 12 to 20	21,014 (3.17)	28,794 (4.75)	47,809 (6.79)	<0.001
Sex				
Female	78,315 (8.93)	91,796 (11.88)	145,951 (16.63)	<0.001
Male	80,831 (9.28)	92,293 (12.17)	147,292 (17.00)	<0.001
Race and ethnicity^b^				
American Indian/Alaskan Native	2,878 (17.22)	3,102 (24.49)	3,701 (25.40)	<0.001
Asian/Pacific Islander	3,177 (7.57)	3,783 (10.83)	6,299 (15.39)	<0.001
Non-Hispanic Black	18,142 (8.34)	17,747 (9.26)	29,432 (13.49)	<0.001
Hispanic	25,126 (5.87)	32,227 (8.70)	44,157 (10.77)	<0.001
Non-Hispanic White	39,766 (10.56)	45,276 (13.40)	71,302 (18.63)	<0.001
Multiracial/Other Race/Unknown	9,123 (8.56)	12,069 (11.92)	21,338 (17.48)	<0.001
Residence designation				
Rural	44,245 (13.75)	48,809 (17.47)	76,319 (23.85)	<0.001
Urban	114,901 (8.05)	135,280 (10.81)	216,924 (15.23)	<0.001
State dental coverage status (D1354 or D1355)				
No coverage	26,427 (2.81)	20,124 (5.00)	3,533 (2.74)	<0.001
Coverage	132,719 (16.44)	163,965 (14.53)	289,710 (17.94)	<0.001

^aBeneficiaries from 5 states were excluded due to data quality concerns.^

^bAnalyses stratified based on race and ethnicity excluded 19 additional states due to data quality concerns.^

^cχ2 tests were used to test statistical significance of utilization rates among categorical variables in 2019, 2020, and 2021.^

**Figure 2:**
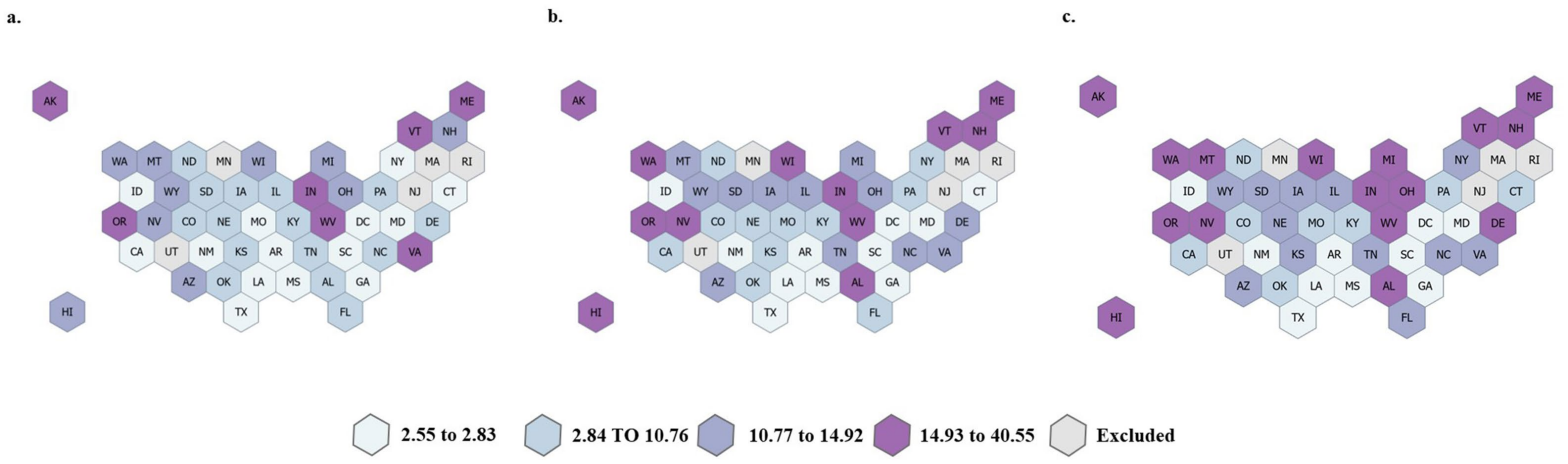
State-level utilization of SDF utilization (D1354 or D1355) per 1,000 Medicaid/CHIP beneficiaries 0–20 years old in **(a)** 2019, **(b)** 2020, and **(c)** 2021. Five states were excluded due to data quality issues.

The SDF utilization demonstrated variation by age, race, and rurality. Utilization was highest among the 0 to 6-year-old age group, male children, American Indian/Alaskan Native children, and children living in rural areas. Children living in rural areas had significantly larger increases in SDF utilization relative to baseline. Many states experienced an increase in SDF utilization; however, some states had similar rates across all time periods, as shown in Figure 2.

The multilevel logistic regression model results (Table 4) noted significant variables predicting the odds of receiving SDF treatment. Compared to children ages 0 to 6, children 7 to 11 were 49% less likely to receive SDF [adjusted odds ratio (aOR): 0.51, 95% confidence interval (CI): 0.50–0.51], and children 12–20 were 83% less likely to receive SDF (aOR: 0.18, 95%CI: 0.17–0.18). Compared to non-Hispanic white children, American Indian/Alaskan Native children were 54% more likely (aOR: 1.54, 95%CI: 1.49–1.60), Asian/Pacific Islander children were 13% more likely (aOR: 1.13, 95%CI: 1.10–1.16), Hispanic children were 11% more likely (aOR: 1.11, 95%CI: 1.10–1.13), and Multiracial children were 2% more likely (aOR: 1.02, 95%CI: 1.00–1.04) to receive SDF. Compared to non-Hispanic white children, Non-Hispanic Black children were 10% less likely to receive SDF (aOR: 0.90, 95%CI: 0.89–0.91). Compared to children with urban residence, children with rural residence were 18% more likely to receive SDF (aOR: 1.18, 95%CI: 1.16–1.19). Most notably, compared to children living in states without SDF coverage, children living in states with SDF coverage were 10.5 times more likely to receive SDF (aOR: 10.55, 95%CI: 3.45–32.24).

**Table 4:** Multilevel logistic regression model predicting the odds of receiving silver diamine fluoride (SDF), 2021.

	aOR (95% CI)^a^
Age Groups (Ref. Age 0 to 6)	
Age 7 to 11	0.509 (0.503–0.514)^b^
Age 12 to 20	0.175 (0.172–0.177)^b^
Sex (Ref. Male)	
Female	0.997 (0.988–1.007)
Race and Ethnicity (Ref. Non-Hispanic White)	
American Indian/Alaskan Native	1.544 (1.488–1.602)^b^
Asian/Pacific Islander	1.128 (1.098–1.159)^b^
Non-Hispanic Black	0.899 (0.886–0.912)^b^
Hispanic	1.111 (1.096–1.126)^b^
Multiracial/Other Race/Unknown	1.018 (1.002–1.035)^b^
Residence Designation (Ref. Urban)	
Rural	1.178 (1.164–1.193)^b^
State SDF Coverage Status (Ref. No Coverage)	
Coverage	10.546 (3.450–32.235)^b^

The model includes the 11,883,341 beneficiaries that live in the 27 states that can be reported because of no claims data and race and ethnicity data quality concerns in 2021.

^aAdjusted odds ratio.^

^bp < 0.05.^

## Discussion

### Summary of results

This multi-year cross-sectional study quantified how the COVID-19 PHE significantly influenced SDF utilization among Medicaid and CHIP enrollees. Pediatric SDF utilization per 1,000 enrollees nearly doubled from 2019 to 2021, while overall dental utilization dropped during 2020 and rebounded to 2019 levels in 2021. SDF utilization had variation by age, rurality, and race/ethnicity. Utilization was highest for children ages 0–6, children living in rural areas, and American Indian/Alaskan Native enrollees. SDF utilization was lowest for children ages > = 12, children living in urban areas, and non-Hispanic Black enrollees. Having a reimbursement policy within the state’s Medicaid program significantly increased SDF utilization.

### Importance of results

This study’s results show how SDF, as an oral health policy strategy, helped solve a public health problem during the COVID-19 PHE. Medicaid reimbursement policy can be a strong driver of dental utilization (16, 17). In this study, children living in states with an SDF reimbursement policy had 10 times the utilization rate of those without a policy. Nearly every state Medicaid program now has a reimbursement policy for D1354 in its fee schedule.

The benefits of SDF treatment stem from its minimally invasive application technique. It does not require local anesthesia, sedation, or general anesthesia, nor does it require handpieces for application (5). It can be applied in the early stages of disease and prevent disease progression, which makes it ideal for young children, individuals with special health care needs, individuals with dental care-related fear and anxiety, and those who live in rural areas (5, 6). When looking at any dental utilization data in general, children under 3 years old and those living in rural areas often have the lowest utilization rates (18–20). The current study results found that children who fall into these subpopulations had the highest rates of SDF utilization. This matches clinical experience and professional guidelines recommending SDF for very young children or when access to care is difficult (6). SDF in community-based settings has allowed children to have their cavities treated locally when otherwise they would have been referred for specialty care (21, 22). As an oral health policy strategy during the PHE, SDF reimbursement seemed to increase access to dental care for young children and those from rural counties. These groups traditionally have a difficult time receiving dental treatment.

Although SDF utilization increased across all age, race/ethnicity, and rurality groups from 2019 to 2021, the increase was not uniform within each variable. Non-Hispanic black children had the smallest increase in SDF utilization rate and were less likely to receive SDF than any other race/ethnicity group. Historically, dental caries experience is greater, and access to dental care is more limited for non-Hispanic black children (2, 23, 24). Because our measure of SDF utilization used a denominator of children who had a dental visit, other factors likely play a role in SDF utilization having a smaller increase for this population. Previous studies have explored the reasons underpinning lower dental service utilization among Black populations. The explanation is multifactorial and multidirectional (meaning both dentist- and patient-perspectives are considered) and includes topics like implicit bias, mistrust in the system, oral health fatalism, and negative previous experiences (25–28). Dentist preferences for restorative treatment and implicit bias could explain comparatively smaller increases in SDF utilization for non-Hispanic black children. While no study has examined dentist preferences or implicit bias specifically for SDF, dentists were more likely to recommend extraction instead of root canal treatment for black patients (29). Parental and cultural preferences against the unesthetic black stain of SDF treated teeth could also explain lower increases in SDF utilization among non-Hispanic black children. However, few studies have examined the association between race/ethnicity or cultural backgrounds and parental acceptance of SDF treatment, and none of these found a relationship between race/ethnicity and parental acceptance of SDF (30–32). Provider and parent education, as well as shared decision-making support tools, could improve equitable access to SDF treatment (33). Public health policies can be designed to support more equitable use of SDF through reimbursement.

### Study shortcomings and strengths

The study has several shortcomings that limit the interpretation. First, the data source was Medicaid claims, which are only as accurate as what providers file for reimbursement. We attempted to account for this by only including states with sufficient data quality, as noted in the Data Quality Atlas (15). Second, by being a multi-year state-level analysis, we did not examine the impact of SDF application on subsequent dental treatment. To determine this impact would require child-level analysis. A recent study identified certain age and tooth thresholds that make subsequent dental treatment more or less likely (34). Third, our multilevel logistic regression only included 1 year of data, so we did not assess the influence of each variable over time. We would expect a washout or lag in SDF claims immediately following the implementation of a new policy, but our data was not structured to assess these influences over time. Fourth, we did not examine cost-effectiveness or dental expenditures. Studies of Medicaid and individual practice data show no significant differences in dental expenditures between children treated with and without SDF after 2 years (35, 36). Fifth, we did not account for clustering around providers. In previous studies, pediatric dentists used SDF more than general dentists in a national claims analysis (37), and provider training likely played a role. In 2015, only 25% of pediatric dentistry residency programs taught and used SDF as part of a caries management plan; in 2020, every program did (38). Early SDF adopters could be more likely to be high users. Last, since Medicaid claims data were the data source, it is likely that SDF treatments were under-reported. Some providers may apply SDF and bill for it under a different CDT code than used in this study, or they use SDF without filing an insurance claim for it as a non-covered service.

The primary strength of this study is its use of national data over 3 years to describe SDF utilization changes during the COVID-19 PHE. Another strength in the study design is the broad inclusion criteria that allowed us to include all non-dually eligible children rather than draw a sample or multiple samples across and within states.

### Future directions

In a shared decision-making framework, the clinical decision to use SDF is likely a combination of numerous clinical, social, cultural, and policy factors across child, family, and dentist levels. These individual decisions about whether to use SDF influence population oral health measures. Future studies are planned to determine the impact of Medicaid SDF reimbursement policies on downstream state-level oral health outcomes such as SDF as a fraction of caries treatment, as well as rates of dental caries treatment, treatment under sedation or general anesthesia, and emergency department visits for non-traumatic dental reasons. Additionally, future work will examine providers’ adoption of SDF and how they consider child- and family-level factors in their clinical decision-making.

## Conclusion

The COVID-19 PHE significantly altered dental practice. States responded by implementing policies supporting minimally aerosolizing treatments such as SDF. As a result, SDF utilization increased each year of the study period, while any dental utilization decreased in 2020 before rebounding to pre-PHE levels in 2021. The findings can be used to develop targeted approaches for clinicians to improve access to SDF treatment to address oral health disparities.

## Data Availability

The raw data supporting the conclusions of this article will be made available through a signed data use agreement with the Centers for Medicare and Medicaid Services. Further inquiries can be directed to the corresponding author.
